# PreserFlo MicroShunt Outcomes in Asia: Two-Year Results with Exploratory Comparisons of Standalone and Combined Surgery from a Regional Tertiary Centre

**DOI:** 10.3390/jcm15207791

**Published:** 2026-10-09

**Authors:** Abdus Samad Ansari, Yamon Thant Syn, Phey Feng Lo, Zhu Li Yap, Paul J. Foster, Shamira A. Perera, Tina T. Wong

**Affiliations:** 1 Singapore National Eye Centre, Singapore 168751, Singapore; ansari.abdus.samad@singhealth.com.sg (A.S.A.); yamon.syn@snec.com.sg (Y.T.S.); lo.phey.feng@singhealth.com.sg (P.F.L.); yap.zhu.li@singhealth.com.sg (Z.L.Y.); paul.foster@singhealth.com.sg (P.J.F.); 2 UCL Institute of Ophthalmology, University College London, London EC1V 9EL, UK; 3 Singapore Eye Research Institute, Singapore 169856, Singapore; 4 Duke-NUS Medical School, Singapore 169857, Singapore; 5 NIHR Moorfields Biomedical Research Centre, Moorfields Eye Hospital NHS Foundation Trust and UCL Institute of Ophthalmology, London EC1V 2PD, UK

**Keywords:** PreserFlo MicroShunt, glaucoma surgery, mitomycin C, surgical outcomes, combined surgery

## Abstract

**Background/Objectives:** To evaluate the two-year efficacy and safety of PreserFlo MicroShunt (PMS) implantation in an Asian cohort with primary open-angle glaucoma via post hoc exploratory comparison of standalone PMS versus combined phacoemulsification and PMS (PMS+Phaco). **Methods:** This prospective single-centre cohort study included 30 consecutive eyes of 30 patients who underwent PMS implantation and were followed for 24 months. Nine eyes underwent standalone PMS and 21 underwent PMS+Phaco. The primary outcome was surgical success assessed using Kaplan–Meier survival analysis. Qualified success was defined as intraocular pressure (IOP) of 6–21 mmHg with ≥20% reduction from baseline, with or without IOP-lowering medication; complete success required no glaucoma medications. Failure was defined as not meeting success criteria at two consecutive visits, with the earliest failure at 3 months. WGA-style definitions were retained to ensure comparison with the published literature. **Results:** Median age was 73 years (IQR 70–78), baseline IOP was 16.0 mmHg (14.0–18.4), and patients were using a median of 3 (2–3) IOP-lowering medications. At 24 months, median IOP was 15.0 mmHg (13.0–17.1), with 57% of eyes remaining medication-free. Whole-cohort qualified success at 24 months was 36.7% (95% CI 20.1–53.4). Qualified success was 44.4% (95% CI 13.6–71.9) for standalone PMS and 33.3% (14.9–53.1) for PMS+Phaco (log-rank *p* = 0.586). Using alternative IOP thresholds, 83.2% of eyes achieved qualified success at IOP < 18 mmHg and 76.5% did so below the cohort baseline mean of 16.5 mmHg. Corneal oedema was more frequent after combined surgery (38.1% vs. 0%). Bleb needling was required in 40% of eyes. **Conclusions:** PMS implantation, performed alone or with phacoemulsification, was associated with sustained reduction in medication burden, with 57% of eyes remaining medication-free at 24 months. Absolute IOP control over 24 months was modest. In post hoc exploratory analysis, no difference in surgical success was detected between procedural approaches; however, confidence intervals were wide and data were insufficient to establish equivalence. Low success rates using conventional criteria likely reflect the cohort’s relatively low baseline IOP (median 16.0 mmHg).

## 1. Introduction

Glaucoma remains a leading cause of irreversible blindness worldwide, with reduction in intraocular pressure (IOP) being the only proven strategy to slow disease progression [1]. While topical medical therapy and laser interventions are effective in many patients, surgical management is required in cases of progressive or inadequately controlled disease [2]. Trabeculectomy is the “gold-standard” filtration procedure, often achieving substantial and lasting IOP reduction, but is associated with significant postoperative morbidity and variability in outcomes related to wound healing responses [3,4], and also requires intensive early follow-up.

The development of subconjunctival drainage devices has sought to provide a more standardised approach to filtration surgery while maintaining effective IOP reduction. The PreserFlo MicroShunt (PMS) is an ab externo implanted device composed of poly(styrene-block-isobutylene-block-styrene) (SIBS), a highly biocompatible material designed to minimise inflammation and fibrosis [5,6]. Early and mid-term studies have demonstrated that PMS implantation results in meaningful reductions in IOP and medication burden [7,8,9]. In a multicentre cohort of 100 eyes, median IOP decreased from 21.5 mmHg preoperatively to 13 mmHg at 12 months, with corresponding reductions in medication use [10]. Similar findings have been reported in comparative studies, with IOP reductions of approximately 5–7 mmHg at 1 year following PMS implantation [11]. Despite these encouraging results, success rates vary substantially depending on the IOP threshold and percentage reduction criteria applied, with more stringent definitions associated with lower apparent success rates [10].

Emerging evidence suggests that outcomes may differ across patient populations; for instance, reduced filtration surgery survival has been reported in African-derived populations, which may reflect differences in conjunctival wound healing and fibrotic response [12]. These factors emphasise the importance of evaluating outcomes in populations where variations in baseline IOP, ocular anatomy, and healing response may influence both the absolute IOP reduction and the interpretation of surgical success [13,14]. Longer-term PMS outcome data in Asian populations remain limited. While PMS may be performed as a standalone procedure or in combination with cataract surgery, data evaluating the impact of combined phacoemulsification on surgical outcomes remain limited. Expert consensus suggests that combined procedures are feasible, but further evidence is required to determine their effect on long-term IOP control and wound healing dynamics [15].

The primary aim of this study was to evaluate the safety and effectiveness of PMS implantation over 24 months in an Asian cohort with POAG. Secondary objectives were to assess surgical success using alternative absolute IOP thresholds and, in a post hoc exploratory analysis, to compare outcomes between standalone PMS and combined phacoemulsification and PMS.

## 2. Materials and Methods

This prospective, single-centre cohort study included consecutive patients undergoing PMS implantation with mitomycin C (MMC) at the Singapore National Eye Centre (SNEC) between September 2021 and April 2024. Clinical data were collected prospectively using standardised case report forms and the institutional electronic medical record system. The study adhered to the tenets of the Declaration of Helsinki and was conducted with approval from the institutional review board (R1704/27/2020). Written informed consent was obtained from all participants.

### 2.1. Participants

Patients were eligible for inclusion if they were aged 21 years or older, had a diagnosis of POAG with documented progression or advanced disease requiring a lower target IOP than could be achieved on maximally tolerated medical therapy, and were considered suitable candidates for filtration surgery. A minimum postoperative follow-up of 3 months was required.

Patients were excluded if they had any other form of glaucoma, including neovascular, uveitic, traumatic disease or prior incisional glaucoma surgery. A total of 30 eyes from 30 patients were included and followed for 24 months.

We included both standalone PMS and combined phacoemulsification with intraocular lens implantation and PMS (PMS+Phaco). The decision to perform combined surgery was made at the discretion of the operating surgeon, with PMS+Phaco offered to phakic patients with visually significant cataract, and standalone PMS performed in pseudophakic or in phakic patients without significant cataract.

### 2.2. Surgical Technique

All surgeries were performed by fellowship-trained glaucoma surgeons using a standardised ab externo approach. A fornix-based conjunctival peritomy was made superiorly, and a deep sub-Tenon’s pocket was dissected posteriorly for 8–10 mm. MMC (0.4 mg/mL)-loaded sponges were placed into the intra-tenon space and then irrigated with 50 mL of normal saline. A 25-gauge needle created a transscleral tunnel entering the anterior chamber about 1 mm posterior to the limbus, and the device was inserted with its fins in the scleral pocket and the distal end in the sub-Tenon’s pocket. Aqueous flow was verified, and the conjunctiva was closed with 10-0 nylon sutures, taking care to ensure the microshunt was flat against the sclera and not kinked. In combined procedures, cataract surgery was performed prior to PMS insertion. Postoperatively, patients received topical levofloxacin for 4 weeks and prednisolone 1% tapered over (3–4) months. Preoperative glaucoma medications were discontinued immediately following surgery and reinitiated as deemed necessary by the consultant. Data collection was performed on day 1, week 1, and at months 1, 3, 6, 12, 18, and 24.

### 2.3. Outcome Measures

The primary outcome was surgical success; two definitions were used, consistent with World Glaucoma Association guidelines and major glaucoma surgical trials [16]. Qualified success was defined as an IOP between 6 and 21 mmHg, with a reduction of at least 20% from baseline, achieved with or without IOP-lowering medications, in the absence of additional glaucoma surgery or loss of light perception. Complete success was defined as meeting this criterion without the use of glaucoma medications. Failure was defined as the absence of success at two consecutive postoperative visits, with the earliest possible failure occurring from 3 months following surgery. This approach was used to minimise misclassification of transient early postoperative IOP fluctuations as treatment failure, in keeping with recommended practice for survival analysis in glaucoma surgery [17].

Secondary outcomes included IOP and percentage IOP reduction from baseline at each postoperative visit, the number of IOP-lowering medications, and best-corrected visual acuity (BCVA) measured in logMAR. Intraoperative and postoperative complications were also recorded alongside the need for further intervention, including the need for bleb needling or further glaucoma surgery. The original study was designed to evaluate the safety and effectiveness of PMS implantation; additional subgroup analysis comparing standalone and combined procedures was not pre-specified.

### 2.4. Additional IOP Thresholds Evaluated

Given the low baseline IOP in this cohort, additional secondary analysis was performed using alternative IOP thresholds. This analysis followed modern glaucoma surgical endpoint guidance, including WGA/EGS reporting principles and the recent AAO Preferred Practice Pattern Committee statement on MIGS endpoints, which supports reporting the cumulative probability of surgical success using Kaplan–Meier analysis and recognises the value of alternative IOP thresholds in aiding interpretation across studies [16,18].

We repeated Kaplan–Meier analysis using IOP thresholds of <21, <18, <16.5, <15, and <12 mmHg. The <16.5 mmHg threshold was derived from the cohort mean baseline IOP and is therefore data-dependent rather than a recognised clinical target; it is presented to indicate the proportion of eyes maintaining IOP below their own preoperative level. For this analysis, qualified success was defined as achieving the specified IOP threshold with or without IOP-lowering medication, while complete success required achieving the threshold without IOP-lowering medication. Failure was defined as not meeting the relevant threshold at two consecutive postoperative visits, with reoperation counted as failure.

### 2.5. Statistical Analysis

Given the sample size, all continuous variables are summarised as median (interquartile range, IQR) and analysed using non-parametric methods; categorical variables are reported as a number (percentage). Between-group comparisons of continuous variables used the Mann–Whitney U test, and categorical variables were compared using Fisher’s exact test.

For within-cohort analyses, postoperative IOP, BCVA and medication count were compared with the baseline using the Wilcoxon signed-rank test. Bonferroni correction was applied to account for multiple comparisons across postoperative time points.

Kaplan–Meier survival analysis was used to estimate the cumulative probability of surgical success, with between-group comparisons performed using the log-rank test. Cox proportional hazards regression was used to estimate hazard ratios with 95% confidence intervals for treatment group and pre-specified covariates, including baseline intraocular pressure, age, sex, and lens status. Eyes were censored at the date of the last attended visit. Any death during the follow-up period was censored at that point and was not counted as a surgical failure. Multivariable analysis was not performed due to the limited number of events. A two-sided *p*-value < 0.05 was considered statistically significant. Analyses were performed using Stata version 18 (StataCorp, College Station, TX, USA).

## 3. Results

Results are presented as baseline characteristics, IOP and medication burden over time, survival analysis using the primary success definition, predictors of failure, alternative IOP thresholds, and visual and safety outcomes. Comparisons between standalone PMS and PMS+Phaco are reported within each section and are post hoc and exploratory.

### 3.1. Patient Characteristics

All thirty eyes were included in the analysis. Two patients in the PMS+Phaco group died during follow-up, after the 12- and 18-month visits, respectively, and were censored, leaving 28 eyes (9 PMS, 19 PMS+Phaco) with 24-month data. Of the 30 patients included, 9 patients (30%) underwent standalone PMS implantation and 21 (70%) underwent combined PMS+Phaco. Median age was 73 years (IQR 70–78) and 23 patients (77%) were male. Median baseline IOP was 16.0 mmHg (14.0–18.4) on a median of 3 (2–3) IOP-lowering medications, and median baseline visual field mean deviation (MD) was −13.2 dB (−16.3 to −9.3). Pre-existing comorbidities included hypertension in 21 patients (70%), diabetes mellitus in 9 (30%), hyperlipidaemia in 16 (53%) and ischaemic heart disease in 7 (23%).

The standalone PMS group was older (median 78 years [75–79] vs. 73 [68–74], *p* = 0.030), was predominantly pseudophakic (1 of 9 phakic [11%] vs. 21 of 21 [100%], *p* < 0.001), was more likely to have undergone previous laser intervention (4 [44%] vs. 0, *p* = 0.005), and was on a greater number of baseline IOP-lowering medications (median 3 [3,4] vs. 2 [2,3], *p* = 0.006). Baseline visual acuity was worse in the PMS+Phaco group (median logMAR 0.4 [0.3–0.6] vs. 0.3 [0.2–0.3], *p* = 0.023), consistent with the visually significant cataract in eyes selected for combined surgery. Median baseline IOP did not differ significantly between groups (17.0 mmHg [15.5–20.5] vs. 16.0 [14.0–17.5], *p* = 0.238). Table 1 presents full baseline characteristics.

### 3.2. Intraocular Pressure

Median IOP fell from 16.0 mmHg (14.0–18.4) at baseline to 7.5 mmHg (5.1–8.0) on the first postoperative day. IOP rose over the first three months before stabilising, with median values between 11.5 and 15.0 mmHg from the third month onwards. At 24 months, the median IOP across the cohort was 15.0 mmHg (13.0–17.1). After Bonferroni correction for eight within-cohort comparisons (adjusted threshold *p* < 0.00625), IOP remained significantly lower than baseline at every timepoint from day 1 through 12 months, but not at 18 months (*p* = 0.035) or 24 months (*p* = 0.038).

**Figure 1:**
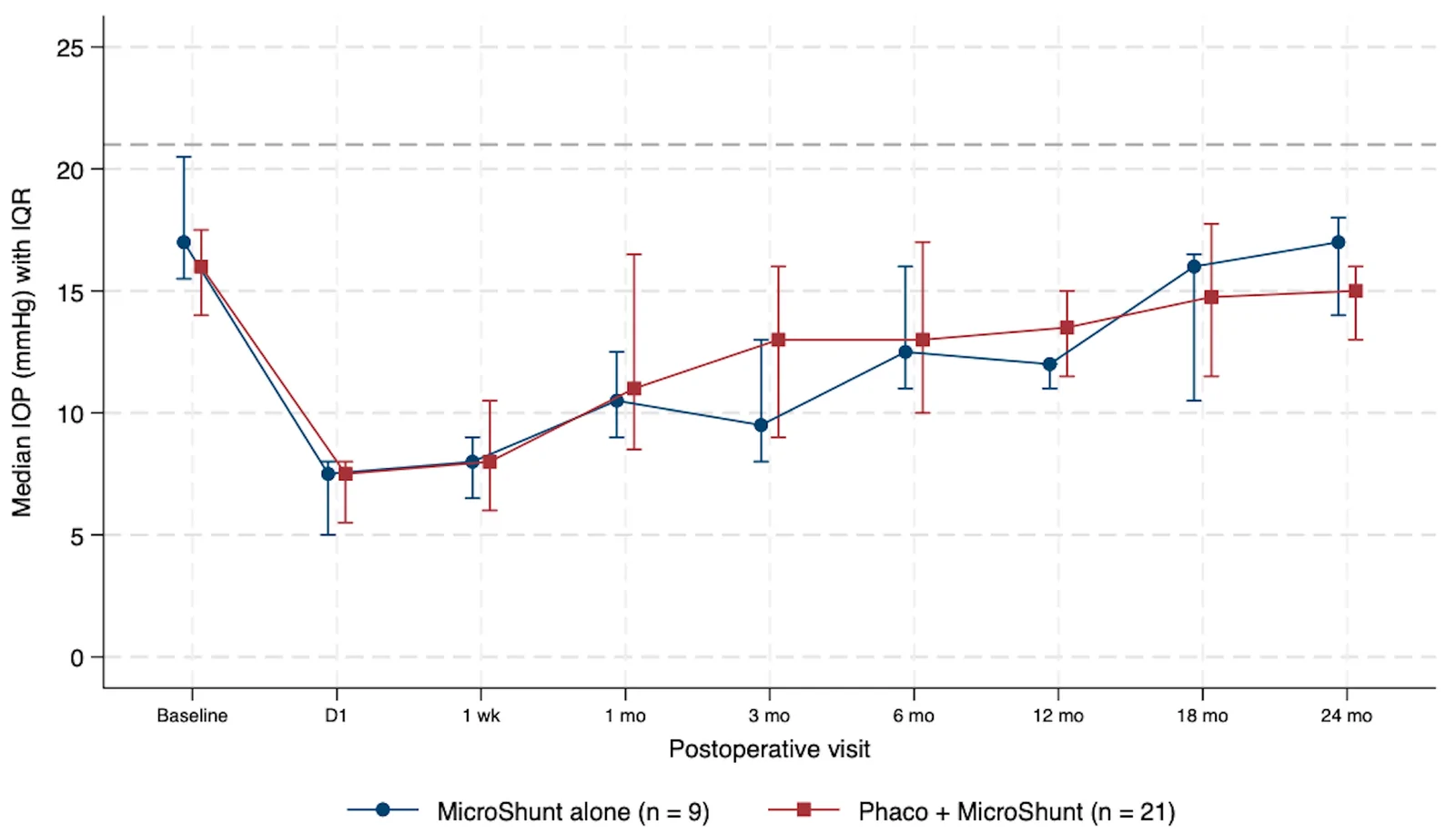
Median IOP at each timepoint for PMS and PMS+Phaco. Error bars show the interquartile range; the dashed line indicates 21 mmHg.

Median IOP did not differ significantly between the two groups at any timepoint (all *p* ≥ 0.172). Percentage IOP reduction from baseline was greater in the standalone PMS group at 12 months (median 38% [23–52] vs. 7% [−12 to 31], *p* = 0.046), with a corresponding difference in absolute reduction (7.5 mmHg [3.5–11.0] vs. 1.0 [−1.5 to 5.5], *p* = 0.023). This difference was not evident at 18 or 24 months. Per-visit IOP and reduction values are presented in Table 2 and Table 3 and Figure 1.

### 3.3. Glaucoma Medication Burden

The median number of IOP-lowering medications fell from 3 (2–3) at baseline to 0 (0–0) at one month, and the median remained 0 at every subsequent visit. After Bonferroni correction for six within-cohort comparisons (adjusted threshold *p* < 0.00833), medication use remained significantly lower than baseline at all postoperative timepoints from 1 month through 24 months (all *p* < 0.001) (Table 4).

All eyes were medication-free at 1 month, falling to 24 of 30 (80%) at 12 months and 16 of 28 (57%) at 24 months. There was no significant difference between groups in medication count or in the proportion remaining medication-free at any timepoint (all *p* ≥ 0.085). Baseline medication burden differed between groups (median 3 [3,4] vs. 2 [2,3], *p* = 0.006).

### 3.4. Survival Analysis

**Figure 2:**
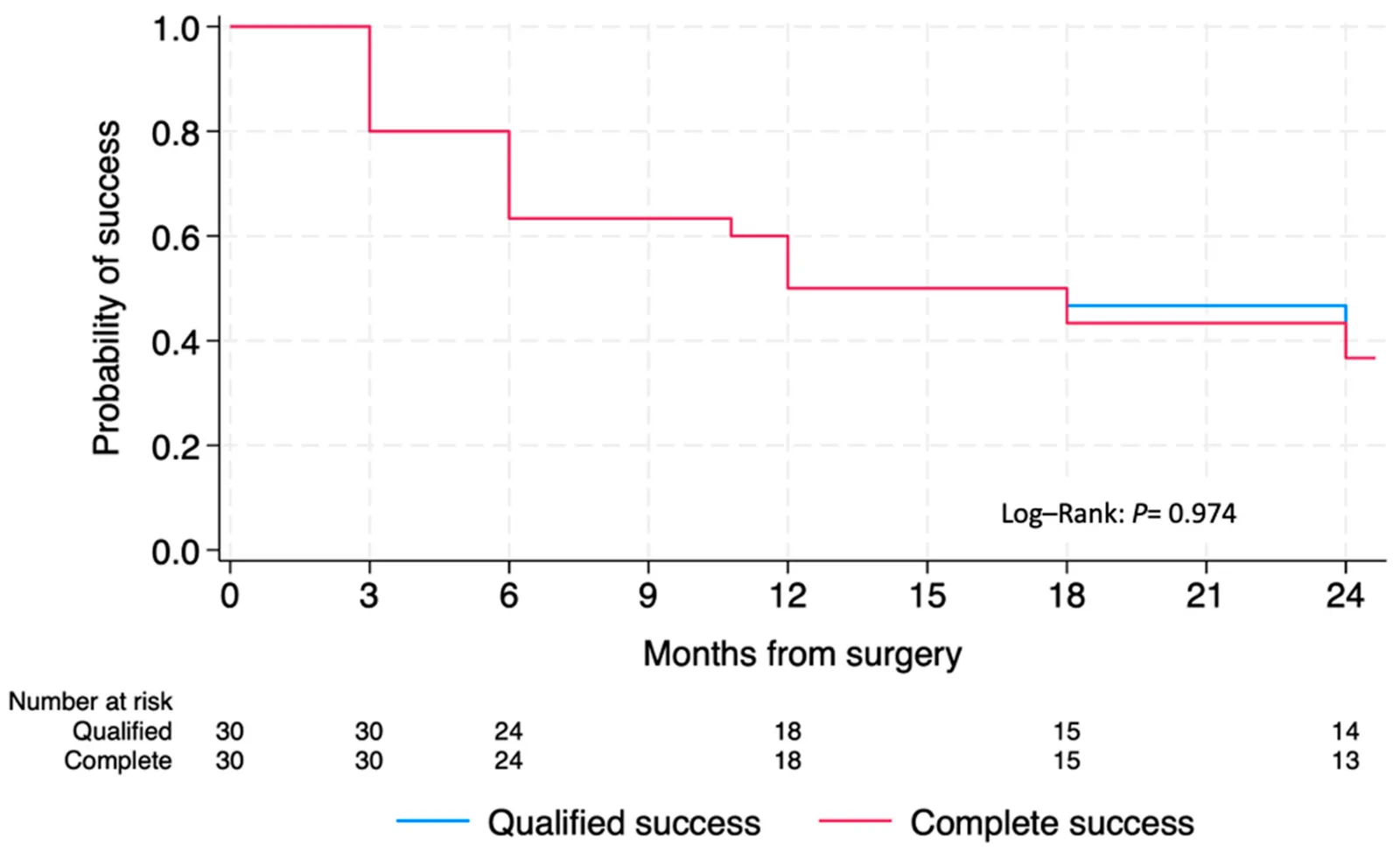
Whole-cohort Kaplan–Meier survival for qualified and complete surgical success.

Over the 24-month follow-up, 19 surgical failures occurred, with identical event counts under both qualified and complete success definitions. Failure required success criteria to be unmet at two consecutive visits, with the earliest possible failure at 3 months. The cumulative probability of qualified success was 80.0% (95% CI 60.8–90.5) at 3 months, decreasing to 63.3% (43.6–77.8) at 6 months, 50.0% (31.3–66.1) at 12 months, and 36.7% (20.1–53.4) at 24 months (Table 5). Curves for complete success closely mirrored those for qualified success, with no meaningful separation between the two definitions (log-rank *p* = 0.974) (Figure 2). The most common reason for failure was an IOP reduction of less than 20% from baseline, despite IOP remaining ≤21 mmHg (n = 15, 78.9%). This was followed by IOP > 21 mmHg (n = 3, 15.8%) and reoperation (n = 1, 5.3%). No eyes failed due to hypotony. Medication reintroduction preceded failure on IOP criteria in one eye, accounting for the only divergence between the qualified and complete success curves.

**Figure 3:**
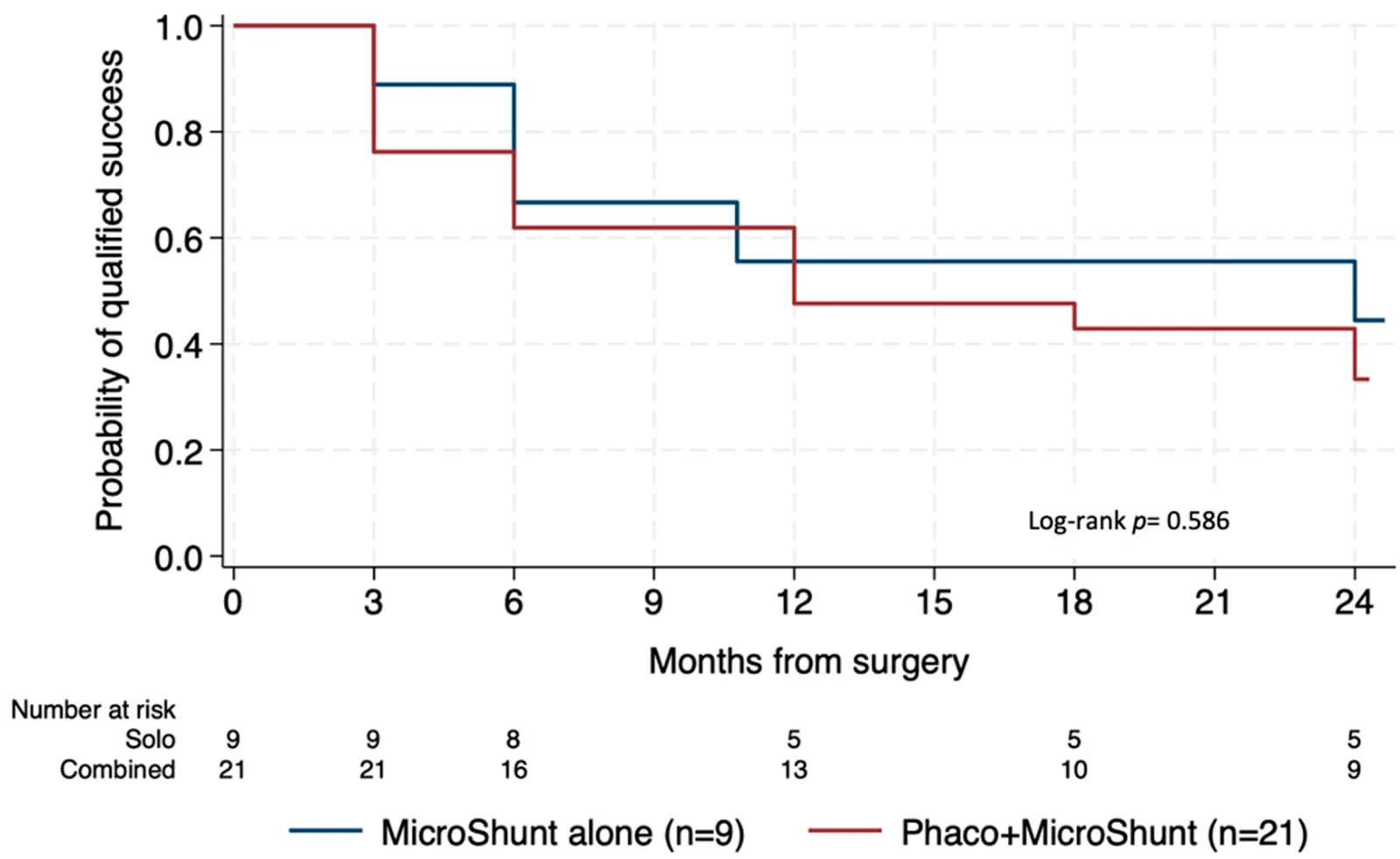
Kaplan–Meier survival for qualified surgical success between PMS and PMS+Phaco.

In subgroup analysis, qualified success at 24 months was 44.4% (13.6–71.9) in the PMS group and 33.3% (14.9–53.1) in the PMS+Phaco group (log-rank *p* = 0.586). A similar pattern was observed for complete success (*p* = 0.577), with no sustained difference between groups over time (Figure 3). Confidence intervals around these estimates were wide and the study was not powered to detect clinically meaningful differences between approaches; the absence of a statistically significant difference should therefore not be interpreted as evidence of equivalence.

### 3.5. Predictors of Failure

Whole-cohort univariable Cox regression for the four pre-specified covariates is shown in Table 6. Age, sex, IOP and lens status were not significantly associated with failure.

### 3.6. Alternative IOP Threshold Analysis

**Figure 4:**
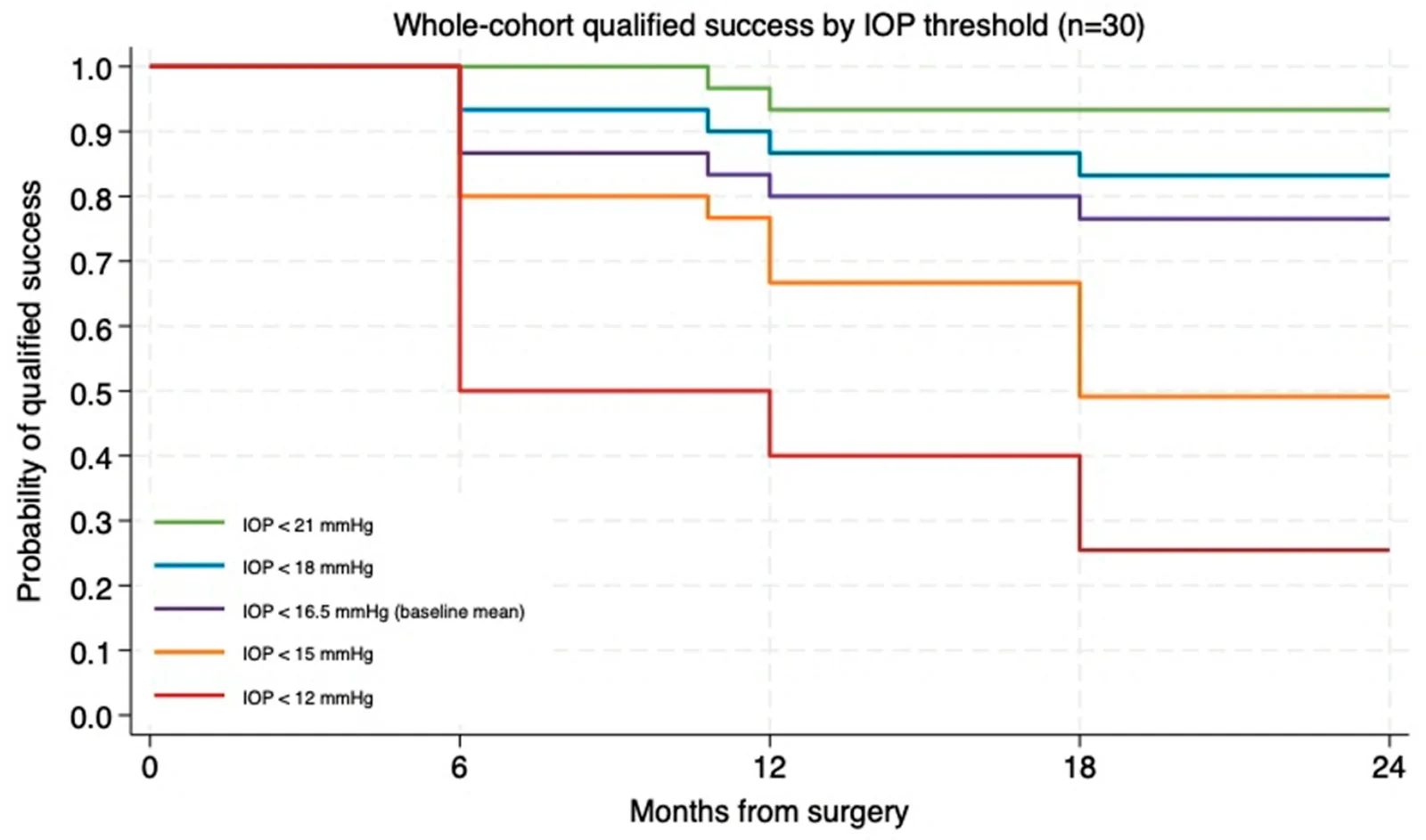
Whole-cohort Kaplan–Meier survival for qualified surgical success at alternative IOP thresholds (<21, <18, <16.5, <15 and <12 mmHg). The 16.5 mmHg threshold is the cohort’s mean baseline IOP.

Given the cohort’s low baseline IOP, secondary Kaplan–Meier analyses were performed using alternative absolute IOP thresholds. At 24 months, whole-cohort qualified success was 93.3% for IOP < 21 mmHg, 83.2% for IOP < 18 mmHg, 76.5% for IOP below the cohort baseline mean of 16.5 mmHg, 49.1% for IOP < 15 mmHg, and 25.5% for IOP < 12 mmHg (Figure 4). Complete success rates at 24 months were 62.9%, 62.9%, 59.4%, 38.8%, and 22.0%, respectively, as shown in Table 7.

In subgroup analyses, there was no statistically significant difference between standalone PMS and PMS+Phaco at any alternative threshold for either qualified or complete success (all log-rank *p* > 0.05), as shown in Table S1: Supplementary Materials.

### 3.7. Visual Outcomes

Median BCVA was logMAR 0.3 (0.2–0.5) at baseline, 0.2 (0.1–0.3) at 12 months and 0.2 (0.2–0.3) at 24 months. After Bonferroni correction for two comparisons (adjusted threshold *p* < 0.025), the improvement from baseline was statistically significant at both 12 months (*p* = 0.002) and 24 months (*p* = 0.010).

At a subgroup level, median BCVA at 24 months was logMAR 0.3 (0.2–0.3) in the PMS group and 0.2 (0.1–0.2) in the PMS+Phaco group (*p* = 0.019). This reflects the expected improvement in the PMS+Phaco group following cataract extraction (median logMAR 0.4 [0.3–0.6] at baseline to 0.2 [0.1–0.2] at 24 months), whereas vision in the PMS group, where the majority of members were pseudophakic at baseline, remained largely unchanged.

### 3.8. Complications and Secondary Procedures

No intraoperative complications were observed. Postoperatively, anterior chamber inflammation was the most frequent finding (n = 18, 60.0%), followed by corneal oedema or folds (n = 8, 26.7%) and hyphaema (n = 6, 20.0%). Bleb needling was performed in 12 eyes (40.0%) during the follow-up period. Of these, 3 (25.0%) maintained qualified success at 24 months, compared with 8 of 18 (44.4%) eyes that did not undergo needling (*p* = 0.442). As needling was performed in eyes with rising IOP, these groups are not directly comparable. Endothelial cell count reduction was documented in 4 patients (13.3%), and hypotony occurred in 3 (10.0%).

Between subgroups, rates of anterior chamber inflammation (55.6% vs. 61.9%, *p* = 0.528), hyphaema (33.3% vs. 14.3%, *p* = 0.329), hypotony (11.1% vs. 9.5%), and needling (33.3% vs. 42.9%, *p* = 0.472) were similar. Corneal oedema or folds were observed more frequently in the PMS+Phaco group (38.1% vs. 0%), consistent with the additional surgical trauma associated with phacoemulsification. Oedema resolved within 2 months in 7 of 8 eyes, and one eye developed corneal decompensation requiring Descemet membrane endothelial keratoplasty at approximately 8 months. Endothelial cell count reduction was also confined to this group (19.0%). This was recorded as a clinical observation; quantitative specular microscopy counts were not systematically collected and the magnitude of cell loss was therefore not reported.

One patient in the PMS group required bleb revision following implant migration at 10.8 months. Subsequently, IOP control remained suboptimal, with the patient later listed for cataract extraction and Ahmed valve implantation at 22 months. No glaucoma-related reoperations occurred in the PMS+Phaco group. Two deaths occurred during follow-up, both in the PMS+Phaco group, and were unrelated to the procedure. Full complication data is presented in Table 8.

## 4. Discussion

In this prospective cohort study, we evaluated 24-month safety and efficacy outcomes following PMS implantation in patients with POAG. Surgical intervention was associated with sustained reductions in medication burden during follow-up, with an acceptable safety profile. Medication burden fell substantially from baseline and remained significantly reduced throughout follow-up, with 57% of eyes remaining medication-free at 24 months. This was of considerable benefit to this cohort given that they were receiving a median of three medications before surgery. In a post hoc exploratory analysis, no significant difference in surgical success was detected between standalone PMS and PMS+Phaco.

The magnitude of IOP reduction observed in this cohort was modest compared with that reported in larger published series; median IOP decreased from 16.0 mmHg at baseline to 15.0 mmHg at 24 months. The corresponding cohort means were 16.5 and 15.1 mmHg, respectively, representing an 8.7% mean reduction. Larger reductions, typically 30–40%, have been reported in cohorts with baseline IOP in the mid-to-high twenties [7,9,19,20]. The lower apparent success rates likely reflect the substantially lower baseline IOP of our cohort (mean 16.5 mmHg) rather than reduced efficacy but may also reflect a possible greater propensity for scarring in Asian populations. The qualified success definition, based on World Glaucoma Association criteria, requires both an absolute IOP threshold and a relative reduction of at least 20% from baseline [16]. In cohorts with lower preoperative IOP, this is inherently more stringent. The identical event counts under the qualified and complete definitions indicate that failure was driven predominantly by the IOP component of the definition rather than by medication reintroduction alone. With a low baseline IOP, the percentage-reduction criterion likely contributed to the declining success curve, as the alternative threshold analysis also suggests, although this cannot be fully distinguished from the loss of bleb function.

The additional alternative IOP threshold analysis provides further context for interpreting these findings. A conventional benchmark requiring ≥20% IOP reduction corresponds to a postoperative IOP of approximately 13.2 mmHg for an eye starting at the cohort mean; this may underestimate clinically meaningful IOP control in eyes, starting in the mid-teens. In contrast, when success was assessed using absolute IOP thresholds, 83.2% of eyes maintained IOP < 18 mmHg and 76.5% maintained IOP below the cohort baseline mean at 24 months, while 59.4% achieved medication-free IOP below the cohort baseline mean. Overall, success ranged from 93.3% at <21 mmHg to 25.5% at <12 mmHg, showing that apparent success in this cohort depends substantially on the threshold applied. These secondary analyses are intended to aid interpretation across studies and do not replace the pre-specified WGA-style definition. This is consistent with contemporary glaucoma surgical reporting guidance, which recognises that alternative success-and-failure criteria using different percentage reductions and upper IOP limits may be presented to aid interpretation across studies.

Outcomes from this cohort should be interpreted in the context of emerging data from similar populations undergoing PMS implantation. Recent series from Japan and other East Asian centres have consistently demonstrated reductions in IOP, with postoperative values typically stabilising in the low teens and relative reductions of 30–40% [21,22]. A large multicentre Japanese cohort has also reported high rates of qualified success at 12 months, often exceeding 70%, particularly in cohorts with higher baseline IOP [23]. In contrast, studies including patients with lower preoperative IOP have reported more modest relative reductions [21]. This suggests that baseline IOP is a key determinant of both the magnitude of IOP reduction and the likelihood of achieving standardised success criteria and should be considered when interpreting surgical outcomes [24,25,26].

To date, prospective data from populations with a case mix comparable to ours remain limited. A single-centre prospective series from a different unit in Singapore represents the only other published study to date [27]. Results demonstrated sustained IOP reduction to the mid-teens, with a corresponding reduction in medication burden over 2 years. Similarly, complete success rates declined over time and were generally lower in combined procedures [27]. Our findings add to this limited Asian evidence base in a cohort with a lower baseline IOP than previously reported, showing that PMS can maintain IOP in the mid-teens with a sustained reduction in medication burden, despite lower success rates under percentage-reduction criteria.

In this post hoc exploratory analysis, we did not detect a sustained difference in outcomes between standalone PMS and PMS+Phaco; however, given the small and unbalanced groups, this should not be taken as evidence that outcomes are equivalent. The difference in IOP at month 12 was not seen at later time points or in the survival analysis and, given the small subgroups and repeated comparisons, is likely to represent a chance finding. Cataracts commonly coexist with glaucoma, and many patients presenting for filtration surgery have visually significant lens opacity [28,29]. Combined procedures offer practical advantages, including a single operative episode and the potential additive IOP-lowering effect of lens extraction. Previous work has demonstrated that combined cataract and filtration procedures can achieve sustained IOP reduction with reduced medication burden in Asian eyes [30]. However, the effect of concurrent cataract surgery on subconjunctival wound healing remains uncertain. Bleb-forming procedures are particularly sensitive to postoperative fibrosis, and the additional inflammatory stimulus from phacoemulsification may influence bleb function and long-term surgical success following PMS implantation [31]. Compared with trabeculectomy, where postoperative manipulations (e.g., argon laser suture lysis, removal of releasable sutures, or bleb massage) can dampen a slowly rising IOP, no such interventions exist for PMS. Thus, the only option is to proceed to needling with antifibrotics much earlier. Nonetheless, trabeculectomy generally achieves lower postoperative IOP and remains the established filtration procedure when very low target pressures are required. PMS offers a different balance of IOP-lowering effects, medication reduction, safety and postoperative intervention.

Our overall complication profile was similar to that reported in previous studies [6,17,27,32]. We observed early postoperative hypotony and transient shallowing of the anterior chamber, with most cases resolving without long-term consequences. Needling was required in 40% of eyes, within the range reported in some Asian PMS series [27,33]. Differences between studies may reflect variation in case mix, surgical technique, antifibrotic use and thresholds for intervention; comparisons across populations and procedures should therefore be interpreted cautiously. There were no differences in complication rates between subgroups, although the study was not designed to detect small variations.

Clinically, the most consistent benefit of PMS in this cohort was a sustained reduction in medication burden, with 57% of eyes medication-free at 24 months. Postoperative IOP was maintained at or marginally below preoperative levels (median 15.0 mmHg at 24 months vs. 16.0 mmHg at baseline), although this difference was not statistically significant at 18 or 24 months after correction for multiple comparisons. PMS may therefore be considered in Asian patients with POAG and relatively low baseline IOP, in whom the principal aim is to reduce medication burden, rather than to achieve substantial further IOP lowering [17,32,34,35,36]. Postoperative bleb needling was required in 40% of eyes, and patients should be counselled that close follow-up and further intervention may be needed. In the absence of adequately powered comparative data, the choice between standalone and combined surgery is currently guided by lens status and visual needs. In this study, allocation to standalone or combined surgery was determined by lens status and cataract severity rather than by randomisation, and the resulting baseline imbalances preclude any causal inference regarding the effect of adding phacoemulsification. Nonetheless, the direction of effect was broadly consistent across the log-rank test, Cox model and alternative IOP thresholds.

This study has several strengths. It represents a prospective evaluation of PMS outcomes in an Asian population with strict procedural uniformity and detailed longitudinal follow-up to 24 months. Including both standalone and combined procedures reflects real-world clinical practice and allows exploratory assessment of the impact of concurrent cataract surgery. In addition, standardised success definitions and survival analysis facilitate comparison with the broader literature. Several limitations should be acknowledged. The small sample size limited statistical power and the precision of subgroup analyses. The choice of surgical approach was non-randomised and affected by baseline characteristics: the standalone group was older, used more baseline IOP-lowering medications, and had more prior laser treatments, which could confound results and introduce selection bias. The cohort’s low baseline IOP might also limit applicability to populations with more advanced disease. No a priori power calculation was performed; therefore, the study was underpowered to detect between-group differences, and the wide confidence intervals around subgroup outcomes should be interpreted accordingly. Additionally, requiring failure at two consecutive visits, with the earliest failure at 3 months, may underestimate clinically relevant early complications, although no eye in this cohort required surgical intervention for hypotony or choroidal effusion within the first three months. Visual field testing was not performed at protocol-defined intervals, so progression could not be assessed; this remains an important outcome for future studies. Lastly, the limited number of events means we did not perform multivariable modelling, which should be considered when interpreting failure predictors. Whilst the protocol was standardised to align with European studies, surgical technique has evolved since recruitment of this cohort, including modifications to implant positioning, peri- and postoperative anti-inflammatory therapy (steroids, ibuprofen), MMC delivery and concentration, and intraoperative technique, including stenting of the PMS and conjunctival fixation. All eyes in this cohort were operated on using the original protocol described in Section 2.2, and none received these later modifications. Outcomes from this historical cohort may therefore not fully reflect those achievable with current surgical practices.

## 5. Conclusions

In the prospective Asian cohort with relatively low baseline IOP, PMS implantation was associated with a sustained reduction in medication burden over 24 months, with 57% of eyes medication-free at the final visit, while IOP was maintained in the mid-teens, although it was not significantly lower than baseline at 18 and 24 months, after corrections for multiple testing. The success rates seen were lower than those previously reported; this is likely partly attributable to the cohort’s lower baseline IOP. While we retained the WGA-style definition for comparability with the literature, alternative absolute IOP thresholds showed that most eyes maintained IOP below 18 mmHg at 24 months. Bleb needling was required in 40% of eyes, emphasising the importance of postoperative surveillance and intervention. Post hoc exploratory subgroup analysis did not demonstrate a sustained difference between standalone and combined procedures, although the study was not powered to establish equivalence. PMS may therefore be a reasonable option for reducing medication dependence while maintaining IOP in Asian patients with POAG and relatively low baseline IOP. Until adequately powered comparisons are available, procedure selection should be guided by lens status and visual needs. Further studies in larger cohorts are required to better define predictors of long-term success and refine outcome definitions in patients with lower baseline IOP.

## Figures and Tables

**Table 1:** Baseline demographics and clinical characteristics.

Variable	Total (n = 30)	PMS (n = 9)	PMS+Phaco (n = 21)
Age (years)	73 (70–78)	78 (75–79)	73 (68–74)
Male	23 (77%)	6 (67%)	17 (81%)
Right eye	12 (40%)	3 (33%)	9 (43%)
Baseline IOP (mmHg)	16.0 (14.0–18.4)	17.0 (15.5–20.5)	16.0 (14.0–17.5)
Baseline MD (dB)	−13.2 (−16.3 to −9.3)	−10.3 (−13.5 to −7.2)	−14.5 (−16.5 to −11.1)
CCT (µm)	524 (508–550)	519 (509–538)	528 (499–557)
Baseline BCVA (logMAR)	0.3 (0.2–0.5)	0.3 (0.2–0.3)	0.4 (0.3–0.6)
Baseline IOP-lowering medications	3 (2–3)	3 (3–4)	2 (2–3)
Phakic	22 (73%)	1 (11%)	21 (100%)
Previous laser surgery	4 (13%)	4 (44%)	0 (0%)
Hypertension	21 (70%)	6 (67%)	15 (71%)
Diabetes mellitus	9 (30%)	3 (33%)	6 (29%)
Hyperlipidaemia	16 (53%)	6 (67%)	10 (48%)
Ischaemic heart disease	7 (23%)	2 (22%)	5 (24%)

Footnote: Continuous variables are median (IQR); categorical variables are n (%). MD, mean deviation; CCT, central corneal thickness; BCVA, best-corrected visual acuity.

**Table 2:** Intraocular pressure (mmHg) at each time point.

Timepoint	Overall (n = 30)	*p*-Value (vs. Baseline)	PMS (n = 9)	PMS+Phaco (n = 21)	*p*-Value
Baseline	16.0 (14.0–18.4)	—	17.0 (15.5–20.5)	16.0 (14.0–17.5)	0.238
Day 1	7.5 (5.1–8.0)	<0.001	7.5 (5.0–8.0)	7.5 (5.5–8.0)	0.802
1 week	8.0 (6.0–9.9)	<0.001	8.0 (6.5–9.0)	8.0 (6.0–10.5)	0.982
1 month	10.8 (8.6–15.5)	0.006	10.5 (9.0–12.5)	11.0 (8.5–16.5)	0.700
3 months	11.5 (9.0–15.4)	0.004	9.5 (8.5–12.0)	13.0 (9.2–15.8)	0.283
6 months	12.8 (10.6–16.8)	0.002	12.5 (11.0–16.0)	13.0 (10.0–17.0)	0.964
12 months	12.0 (11.1–14.9)	0.003	12.0 (11.0–12.0)	13.5 (11.5–15.0)	0.172
18 months	15.0 (11.0–17.0)	0.035	16.0 (10.5–16.5)	14.8 (11.8–17.4)	0.962
24 months	15.0 (13.0–17.1)	0.038	17.0 (14.0–18.0)	15.0 (13.0–16.0)	0.200

Footnotes: Values are median (IQR). Comparison with baseline by Wilcoxon signed-rank test; between-group comparison by Mann–Whitney U test.

**Table 3:** Absolute and percentage IOP reduction from baseline at each time point.

Timepoint	Absolute Reduction (mmHg)			*p*-Value	Percentage Reduction (%)			*p*-Value
	Overall	PMS	PMS+Phaco		Overall	PMS	PMS+Phaco	
Day 1	9.2 (7.0–11.9)	11.5 (7.5–15.5)	8.5 (7.0–11.0)	0.257	56 (46–74)	60 (48–74)	56 (46–66)	0.541
1 week	7.8 (4.8–11.6)	8.0 (6.0–14.5)	7.0 (3.5–9.0)	0.181	47 (34–61)	56 (43–68)	46 (25–60)	0.330
1 month	4.5 (0.5–9.6)	6.5 (4.5–10.0)	4.0 (−3.0 to 7.5)	0.174	29 (2–50)	42 (26–50)	22 (−25 to 48)	0.277
3 months	4.8 (−1.1 to 9.0)	9.0 (4.0–10.8)	4.0 (−1.8 to 7.2)	0.093	33 (−7 to 51)	50 (26–53)	25 (−12 to 40)	0.157
6 months	3.5 (−0.2 to 7.8)	4.5 (2.5–8.0)	2.0 (−0.5 to 7.0)	0.341	19 (−2 to 38)	29 (12–32)	13 (−3 to 38)	0.469
12 months	3.2 (−0.4 to 7.0)	7.5 (3.5–11.0)	1.0 (−1.5 to 5.5)	0.023	23 (−3 to 37)	38 (23–52)	7 (−12 to 31)	0.046
18 months	2.5 (−0.5 to 5.0)	5.0 (0.0–6.0)	2.0 (−0.6 to 4.6)	0.247	15 (−5 to 27)	24 (0–32)	11 (−5 to 24)	0.358
24 months	2.0 (−0.6 to 4.6)	−0.5 (−2.5 to 5.0)	2.0 (1.0–4.5)	0.712	13 (−5 to 26)	−2 (−16 to 23)	13 (6–26)	0.658

Footnotes: Values are median (IQR). Comparison with baseline by Wilcoxon signed-rank test; between-group comparison by Mann–Whitney U test.

**Table 4:** Number of IOP-lowering medications at each time point.

Timepoint	Overall	*p*-Value (vs. Baseline)	PMS	PMS+Phaco	*p*-Value
Baseline	3 (2–3)	-	3 (3–4)	2 (2–3)	0.006
1 month	0 (0–0)	<0.001	0 (0–0)	0 (0–0)	-
3 months	0 (0–0)	<0.001	0 (0–0)	0 (0–0)	0.603
6 months	0 (0–0)	<0.001	0 (0–0)	0 (0–0)	0.906
12 months	0 (0–0)	<0.001	0 (0–0)	0 (0–1)	0.085
18 months	0 (0–1)	<0.001	0 (0–0)	0 (0–1)	0.718
24 months	0 (0–1)	<0.001	0 (0–1)	0 (0–1)	0.956

Footnote: Values are median (IQR). Comparison with baseline by Wilcoxon signed-rank test; between-group comparison by Mann–Whitney U test.

**Table 5:** Kaplan–Meier cumulative probability of surgical success at each timepoint.

Timepoint	Whole-Cohort Qualified Success	Qualified Success		Complete Success	
		PMS	PMS+Phaco	PMS	PMS+Phaco
3 months	80.0% (60.8–90.5)	88.9% (43.3–98.4)	76.2% (51.9–89.3)	88.9% (43.3–98.4)	76.2% (51.9–89.3)
6 months	63.3% (43.6–77.8)	66.7% (28.2–87.8)	61.9% (38.1–78.8)	66.7% (28.2–87.8)	61.9% (38.1–78.8)
12 months	50.0% (31.3–66.1)	55.6% (20.4–80.5)	47.6% (25.7–66.7)	55.6% (20.4–80.5)	47.6% (25.7–66.7)
18 months	46.7% (28.4–63.0)	55.6% (20.4–80.5)	42.9% (21.9–62.3)	55.6% (20.4–80.5)	38.1% (18.3–57.8)
24 months	36.7% (20.1–53.4)	44.4% (13.6–71.9)	33.3% (14.9–53.1)	44.4% (13.6–71.9)	33.3% (14.9–53.1)

**Table 6:** Univariable Cox proportional hazards regression for all pre-specified covariates.

Covariate	Qualified HR (95% CI)	*p*-Value	Complete HR (95% CI)	*p*-Value
PMS+Phaco (vs. PMS)	1.30 (0.47–3.61)	0.615	1.31 (0.47–3.64)	0.605
Baseline IOP (per mmHg)	0.88 (0.76–1.01)	0.073	0.88 (0.77–1.01)	0.072
Age (per year)	0.95 (0.88–1.04)	0.274	0.95 (0.88–1.04)	0.272
Female sex (vs. male)	1.20 (0.43–3.32)	0.731	1.19 (0.43–3.30)	0.739
Pseudophakic (vs. phakic)	0.64 (0.21–1.94)	0.434	0.64 (0.21–1.93)	0.426

**Table 7:** Whole-cohort qualified and complete success by alternative IOP thresholds.

IOP Threshold	Qualified Success: Month 24	Complete Success: Month 24
IOP < 21 mmHg	93.3% (75.9–98.3)	62.9% (43.0–77.5)
IOP < 18 mmHg	83.2% (64.2–92.6)	62.9% (43.0–77.5)
IOP < 16.5 mmHg (cohort baseline mean)	76.5% (57.0–88.1)	59.4% (39.6–74.5)
IOP < 15 mmHg	49.1% (30.3–65.5)	38.8% (21.6–55.8)
IOP < 12 mmHg	25.5% (11.5–42.1)	22.0% (9.2–38.3)

**Table 8:** Complications and secondary procedures.

Complication/Intervention	PMS	PMS+Phaco	*p*-Value
Intraoperative complications	0 (0.0%)	0 (0.0%)	—
Anterior chamber inflammation	5 (55.6%)	13 (61.9%)	0.528
Corneal oedema/folds	0 (0.0%)	8 (38.1%)	0.035
Hyphaema	3 (33.3%)	3 (14.3%)	0.329
Hypotony	1 (11.1%)	2 (9.5%)	0.672
Shallow anterior chamber	0 (0.0%)	2 (9.5%)	0.483
Epithelial erosions	0 (0.0%)	1 (4.8%)	0.700
Endothelial cell count reduction	0 (0.0%)	4 (19.0%)	0.287
Any bleb needling during follow-up	3 (33.3%)	9 (42.9%)	0.472
Secondary filtration procedure	1 (11.1%)	0 (0.0%)	0.300
Death during follow-up	0 (0.0%)	2 (9.5%)	0.483

## Data Availability

The data that support the findings of this study are available from the corresponding author upon reasonable request. The data are not publicly available due to patient confidentiality and institutional ethical restrictions.

## Supplementary Materials

The following supporting information can be downloaded at: https://www.mdpi.com/article/10.3390/jcm15207791/s1, Table S1: Kaplan–Meier cumulative probability of complete success at alternative IOP thresholds at 24 months, stratified by standalone PMS and PMS+Phaco.
