# Supplementary material for: PreserFlo MicroShunt Outcomes in Asia: Two-Year Results with Exploratory Comparisons of Standalone and Combined Surgery from a Regional Tertiary Centre
Source: J Clin Med. 2026 Oct 9;15(20):7791. doi: 10.3390/jcm15207791 (PMC13705064; doi:10.3390/jcm15207791)
Supplement: Supplementary file 1 [file jcm-15-07791-s001.zip › jcm-4559955-supplementary.pdf]

Supplementary Materials

Table S1. Kaplan–Meier cumulative probability of complete success at alternative IOP thresholds at 24 months, stratified by standalone PMS and PMS+Phaco.

Qualified Success: Subgroups by alternative thresholds

IOP threshold	Group	Events / N	Month 6	Month 12	Month 18	Month 24	Log-rank p
**IOP < 21 mmHg**	PMS	1 / 9	100.0% (100.0–100.0)	88.9% (43.3–98.4)	88.9% (43.3–98.4)	88.9% (43.3–98.4)	0.508
	PMS+Phaco	1 / 21	100.0% (100.0–100.0)	95.2% (70.7–99.3)	95.2% (70.7–99.3)	95.2% (70.7–99.3)	
**IOP < 18 mmHg**	PMS	2 / 9	100.0% (100.0–100.0)	88.9% (43.3–98.4)	77.8% (36.5–93.9)	77.8% (36.5–93.9)	0.652
	PMS+Phaco	3 / 21	90.5% (67.0–97.5)	85.7% (62.0–95.2)	85.7% (62.0–95.2)	85.7% (62.0–95.2)	
**IOP < 16.5 mmHg (cohort baseline)**	PMS	2 / 9	100.0% (100.0–100.0)	88.9% (43.3–98.4)	77.8% (36.5–93.9)	77.8% (36.5–93.9)	0.858
	PMS+Phaco	5 / 21	81.0% (56.9–92.4)	76.2% (51.9–89.3)	76.2% (51.9–89.3)	76.2% (51.9–89.3)	
**IOP < 15 mmHg**	PMS	4 / 9	100.0% (100.0–100.0)	88.9% (43.3–98.4)	55.6% (20.4–80.5)	55.6% (20.4–80.5)	0.432
	PMS+Phaco	11 / 21	71.4% (47.2–86.0)	57.1% (33.8–74.9)	46.8% (24.7–66.1)	46.8% (24.7–66.1)	
**IOP < 12 mmHg**	PMS	6 / 9	66.7% (28.2–87.8)	44.4% (13.6–71.9)	33.3% (7.8–62.3)	33.3% (7.8–62.3)	0.497
	PMS+Phaco	16 / 21	42.9% (21.9–62.3)	38.1% (18.3–57.8)	21.8% (7.1–41.6)	21.8% (7.1–41.6)	

Complete Success: Subgroups by alternative thresholds

IOP threshold	Group	Events / N	M6	M12	M18	M24	Log-rank p

**IOP < 21 mmHg**	PMS	4 / 9	100.0% (100.0–100.0)	88.9% (43.3–98.4)	55.6% (20.4–80.5)	55.6% (20.4–80.5)	0.756
	PMS+Phaco	7 / 21	90.5% (67.0–97.5)	66.7% (42.5–82.5)	66.7% (42.5–82.5)	66.7% (42.5–82.5)	
**IOP < 18 mmHg**	PMS	4 / 9	100.0% (100.0–100.0)	88.9% (43.3–98.4)	55.6% (20.4–80.5)	55.6% (20.4–80.5)	0.791
	PMS+Phaco	7 / 21	85.7% (62.0–95.2)	66.7% (42.5–82.5)	66.7% (42.5–82.5)	66.7% (42.5–82.5)	
**IOP < 16.5 mmHg (cohort baseline)**	PMS	4 / 9	100.0% (100.0–100.0)	88.9% (43.3–98.4)	55.6% (20.4–80.5)	55.6% (20.4–80.5)	0.974
	PMS+Phaco	8 / 21	76.2% (51.9–89.3)	66.7% (42.5–82.5)	61.5% (37.6–78.6)	61.5% (37.6–78.6)	
**IOP < 15 mmHg**	PMS	6 / 9	100.0% (100.0–100.0)	88.9% (43.3–98.4)	33.3% (7.8–62.3)	33.3% (7.8–62.3)	0.668
	PMS+Phaco	12 / 21	66.7% (42.5–82.5)	47.6% (25.7–66.7)	42.3% (21.3–62.0)	42.3% (21.3–62.0)	
**IOP < 12 mmHg**	PMS	7 / 9	66.7% (28.2–87.8)	44.4% (13.6–71.9)	22.2% (3.4–51.3)	22.2% (3.4–51.3)	0.687
	PMS+Phaco	16 / 21	42.9% (21.9–62.3)	33.3% (14.9–53.1)	22.2% (7.4–42.0)	22.2% (7.4–42.0)	
